# Osteoarthritis as an Umbrella Term for Different Subsets of Humans Undergoing Joint Degeneration: The Need to Address the Differences to Develop Effective Conservative Treatments and Prevention Strategies

**DOI:** 10.3390/ijms232315365

**Published:** 2022-12-06

**Authors:** David A. Hart

**Affiliations:** Department of Surgery, Faculty of Kinesiology, McCaig Institute for Bone & Joint Health, University of Calgary, Calgary, AB T2N 4N1, Canada; hartd@ucalgary.ca

**Keywords:** osteoarthritis, osteoarthritis subsets, conservative OA care, females, males, OA prevention

## Abstract

Osteoarthritis (OA) of joints such as the knee and hip are very prevalent, and the number of individuals affected is expected to continue to rise. Currently, conservative treatments after OA diagnosis consist of a series of increasingly invasive interventions as the degeneration and pain increase, leading very often to joint replacement surgery. Most interventions are focused on alleviating pain, and there are no interventions currently available that stop and reverse OA-associated joint damage. For many decades OA was considered a disease of cartilage, but it is now considered a disease of the whole multi-tissue joint. As pain is the usual presenting symptom, for most patients, it is not known when the disease process was initiated and what the basis was for the initiation. The exception is post-traumatic OA which results from an overt injury to the joint that elevates the risk for OA development. This scenario leads to very long wait lists for joint replacement surgery in many jurisdictions. One aspect of why progress has been so slow in addressing the needs of patients is that OA has been used as an umbrella term that does not recognize that joint degeneration may arise from a variety of mechanistic causes that likely need separate analysis to identify interventions unique to each subtype (post-traumatic, metabolic, post-menopausal, growth and maturation associated). A second aspect of the slow pace of progress is that the bulk of research in the area is focused on post-traumatic OA (PTOA) in preclinical models that likely are not clearly relevant to human OA. That is, only ~12% of human OA is due to PTOA, but the bulk of studies investigate PTOA in rodents. Thus, much of the research community is failing the patient population affected by OA. A third aspect is that conservative treatment platforms are not specific to each OA subset, nor are they integrated into a coherent fashion for most patients. This review will discuss the literature relevant to the issues mentioned above and propose some of the directions that will be required going forward to enhance the impact of the research enterprise to affect patient outcomes.

## 1. Introduction

### 1.1. Background

Osteoarthritis (OA) is a degenerative disease of joints (knee, hip, spine, ankle, fingers, etc.) that affects mobility, leads to disability, and affects the quality of life for >250 million individuals worldwide (discussed in [1,2]) and an estimated >50 million patients in the USA (~20% of the adult population) [3]. Given that individuals in many countries are living longer, the number of people affected worldwide is expected to double by 2040. Until the last few decades, OA was commonly thought of as a disease of cartilage as that is the tissue that is most affected by the disease processes. Cartilage is aneural and avascular, so it is compromised when self-healing is considered (discussed in [4]). However, OA is now considered a disease of the whole joint [5,6], and thus, the multitude of components that make up a joint such as the knee, are involved [cartilage, ligaments, tendons, menisci, synovium, capsule and muscle].

While the term osteoarthritis implies that the degenerative process involves inflammation, for many years it was considered solely a degenerative process ascribed to aging-related “wear and tear” or due to injury that disturbed the relationship between the components of a joint leading to the demise of the “weakest link”-cartilage [7]. However, it is now fairly clear that inflammatory processes are involved in OA progression [4,8,9,10,11,12], but it is not clear whether inflammation is part of the initiation process leading to overt OA in different subsets of the condition.

### 1.2. Purpose of the Review

The purpose of this review is to discuss emerging aspects regarding the different subtypes of OA, focusing on the knee, and how the investigation of subtypes of OA may provide clues to the mechanistic basis for the conditions. Such investigations will potentially have three important outcomes: (1) the possibility to identify subtype unique mechanisms that will guide the development of effective interventions to stop disease progression early after diagnosis; (2) the identification of risk factors leading to the development of OA and thus, preventive measures; and (3) development of subtype-specific platforms for integrative conservative care of patients to eventually complement effort # 1. This review will attempt to address and synthesize the way forward using this information and the results of a research effort that is more translation-focused rather than discovery using models not clearly related to human OA.

The material that was reviewed for this article came from PubMed using search terms that included osteoarthritis (>100,000 items), osteoarthritis and human, knee osteoarthritis, knee osteoarthritis and human, knee osteoarthritis and arthroplasty, knee osteoarthritis and conservative care, post-traumatic osteoarthritis, osteoarthritis and epigenetics, osteoarthritis and menopause, osteoarthritis and treatments, osteoarthritis and pain, as well as those search terms and reviews. While attempts were made to focus on recent articles (past 5 years), some citations are of original studies that remain relevant to the discussion.

## 2. Subsets of Knee Osteoarthritis

### 2.1. Background

While OA can affect many joints (i.e., knee, hip, shoulder, elbow, ankle), a large percentage of patients have OA of the knee and hip [3]. As such, patients often experience variable levels of pain and disability, and much research and clinical trial effort have focused on hip and knee OA. However, after spending billions of research dollars and much effort over the past decades, there are currently no effective treatments to stop disease progression in the broad base of patients, and certainly, no treatments that can reverse the joint damage incurred as a consequence of the OA are available [10,11]. Such lack of progress has been discouraging for both the millions of patients with the disease, as well as companies that have invested heavily in research programs. Such frustration by patients can lead to them searching the internet for potential treatments [13], even if not scientifically proven and validated. Why progress has been so slow is likely due to a multitude of factors, such as under-appreciation of the complexity of the problem, the use of preclinical models that do not accurately reflect human disease, a failure to address the mechanistic variation in different subsets of patients, a lack of appreciation of the contribution of inflammatory processes in disease progression, disconnects between biologists and biomechanical experts, and an inability to determine when the condition actually is initiated for most patients other than those with post-traumatic OA (PTOA) [11]. Some of the potential subtypes of knee OA are summarized in Table 1. Table 1 also includes a subtype labeled “Idiopathic,” which contains those patients that cannot be categorized currently. However, with the concept of distinct potential subtypes, the number of patients in the idiopathic category will be small, and research on the care and treatment of those that can be categorized should lead to more precision-oriented outcomes. Of note, nearly all subtypes of OA have inflammatory components that develop during disease progression, but it remains to be determined whether inflammation is intimately involved with disease initiation.

This concept that OA is heterogeneous [14,15] and that sub-typing or phenotyping of OA is needed has also been raised by others [16,17,18,19,20] using a variety of criteria. Thus, some are related to metabolic phenotype [16,18,20], while others rely on patient characteristics at other levels [17,19]. In addition, biomarkers may be of use to identify subsets of patients with unilateral knee OA who may progress to bilateral knee OA [21].

It should also be noted that patients with OA usually present with pain. However, there is only a modest correlation between structural alterations as detected by X-rays or MRI and pain [22,23]. Pain severity can increase during disease progression, possibly due to epigenetic alterations [24]. In fact, approximately 20% of patients receiving joint arthroplasty for their OA have persistent pain after the surgery for mainly unknown reasons [25,26,27]. Due to the complexity of the interface between pain, chronic pain and persistent pain and the structural alterations associated with OA progression and treatment, this review will focus on subtypes of knee OA but not on the correlations with pain or pain mechanisms.

### 2.2. A Focus on Knee Osteoarthritis Associated with Knee Injury (Post-Traumatic Osteoarthritis; PTOA)

Knee OA can arise in some patients after an injury to the knee (such as a meniscal tear or an anterior cruciate ligament (ACL) rupture). While post-traumatic osteoarthritis constitutes ~12% of human knee OA patients [11,28], the vast majority of preclinical research is performed with PTOA models and most is performed with small animal models such as mice [29,30] which yield scientifically interesting results (mainly biological) but based on the low rate of progress, little clinical relevance. Some research has been performed with larger preclinical models such as sheep [31] or pigs or others [32,33], which has provided some more interesting mechanical outputs but again has primarily used post-traumatic OA approaches. In spite of this limitation, the use of large animal models has reinforced the fact that patient heterogeneity is a critical issue that needs to be faced, particularly in the design of clinical trials. That is, the response of each sheep to an ACL transection or some other knee injury is unique [34,35,36], and thus making group comparisons (as is done in randomized, double-blind clinical trials) does not capture the need to address individual variation. The suggestion has been that the *n* = 1 design may be a better option [34,35,36].

In PTOA, the critical element that has been lost is the biomechanical integrity of the joint following injury or rupture to a specific component of the joint. As the joint is considered a multicomponent organ system [5,6], loss of function for one component would disrupt the integrity of the whole organ system. This could not be corrected solely by biological interventions, and a return to mechanical stability is also required. Interestingly, after rupture of the anterior cruciate ligament (ACL), a key contributor regarding knee function, even if the ACL is reconstructed to provide some mechanical stability, ~50% of such individuals will still develop OA by 10 years post-surgery [37,38,39,40]. Why 50% are at risk and 50% at lower risk to develop OA is not known, but it may involve the functioning of muscles around the knee as it is known that there can be a significant loss of quadricep muscle function after tearing the ACL in a subset of such patients [41,42], and this can persist after ACL reconstruction [43,44]. Another variable that appears to influence post-ACL rupture is the placement of the ACL graft during reconstruction [45].

Alternatively, the risk of developing knee OA (or in other joints) following an injury could be due to the persistence of an inflammatory response. Injury to a joint tissue, such as an ACL rupture, will initiate an injury response led by inflammation which can influence a variety of joint tissues [46]. Subsequent surgery to reconstruct the ACL would be another insult to the joint, leading to further inflammation. Persistence of such inflammation could lead to the loss of additional joint tissue integrity and the development of OA-like disease. Interestingly, in preclinical models, administration of anti-inflammatory drugs such as corticosteroids immediately after PTOA-inducing surgery can diminish the inflammation and progression to OA-like disease in sheep and rabbits [47,48,49,50], as well as pigs [51]. Some uptake of this concept has occurred in the clinical literature as well (discussed in [4]). Certainly, such a catabolic and inflammatory environment should be controlled if other interventions such as stem cell injections, tissue-engineered construct implantation, or the use of anabolic interventions are contemplated [4,28]. However, controlling inflammation alone will not address the mechanical dysfunction in PTOA, so a multimodal approach is needed.

If only 12% of human knee OA is due to PTOA, that means other initiating mechanisms must be entertained to address the remaining ~88% of cases. Some other options for potential initiators of knee OA include metabolic dysregulation such as that accompanying obesity, a post-menopausal phenotype to account for the increase of non-PTOA incidence in the post-menopausal female population, variation in lower extremity set points that arise during growth and maturation that are impacted by the aging process (discussed in [4,28]), and of course, genetic and epigenetic factors that could be independent contributors to risk for OA development or contribute to one of the previously mentioned options.

### 2.3. Metabolic OA

Metabolic OA is the term used to describe the development of knee OA in conjunction with obesity [52,53,54]. Individuals with obesity are at increased risk of developing knee OA [55,56]. While some have suggested the development of knee OA in patients with obesity is due to the increased mechanical load on the joint, others have suggested it is due primarily to the metabolic dysfunctions that arise as a consequence of obesity [55]. Thus, many patients that are overweight or have overt obesity develop a cadre of metabolic changes labeled “metabolic syndrome” [57]. Included are conditions such as low-grade systemic inflammation, the risk for type II diabetes, and others [57]. Thus, this interpretation of the risk for knee OA development is a consequence of obesity and not strictly a direct biomechanical effect on the joint. As such, it should be possible to develop interventions that inhibit or prevent the development of the consequences of obesity, even if weight loss is not achievable. Often patients with obesity and knee OA are told to lose weight, but that directive is not readily fulfilled by many patients.

Recent studies in a rat model of diet-induced obesity development with the concurrent development of knee and shoulder joint damage with less evident hip damage have been reported [58]. Interestingly, aerobic exercise or concurrent administration of a prebiotic (commercially available inulin preparation) both prevented the development of diet-induced joint damage [59] as well as some of the metabolic sequelae of the obesity-inducing diet [59]. In this rat model, the results led to the conclusion that the prebiotic was impacting the microbiome of animals that had been altered by the high-fat, high-sucrose diet. As a result of those encouraging results, a clinical trial of the prebiotic in knee OA patients with obesity has been undertaken [60]. This trial was also formulated based on previous results with non-OA patients with obesity. The positive results of this trial (manuscript in preparation) lend support to using prebiotics along with other interventions for the conservative treatment of OA patients.

A key potential aspect of the metabolic syndrome that could be inhibited by prebiotic treatment is the inflammatory component which is emerging as a common element of OA (i.e., OA is an inflammatory disease) [52,53,61]. Secondly, prebiotic treatment of those with knee OA and obesity may be one “tool” in the toolbox for clinical interventions, but one that should be considered in the context of a multimodality treatment regimen.

### 2.4. Post-Menopausal Onset Knee OA in Females

As discussed previously [11,62], the incidence of knee OA in the years prior to menopause in females and males is ~1/1, but after menopause, the ratio increases to >2/1 (F/M). Thus, after menopause, a subset of females now develop OA, which has been categorized as “idiopathic” but may represent a unique subset of patients.

Clearly, hormones can influence the integrity of the connective tissues of the knee [63]. For instance, knee laxity in a significant subset of young females varies across the menstrual cycle [64]. However, ~20% of females in the studies did not vary for unknown reasons, but this finding does indicate females are heterogeneous with regard to responsiveness to hormone variation. Joint laxity can also vary during pregnancy in many females [65], again indicating a potential hormonal influence on joint integrity. As all connective tissues of knee joint tissues express estrogen and progesterone receptors and respond to hormones [63,66,67], this means that all such tissues could be affected by a loss of hormones associated with menopause. However, only a subset of post-menopausal females progress to develop OA, and therefore, there must be some unique features in such females that pose the OA risk.

There are a number of factors that could contribute to OA risk in such females. There may be a genetic risk that is only evident following the loss of hormones after decades of menstrual cycles following the onset of puberty. This may relate to the biology of the extracellular matrix components or even to genetic variation in hormone receptors such as those for estrogen (i.e., ER-alpha and ER-beta). There could also have been the induction of epigenetic changes occurring during the lifespan that could enhance risk after menopause for a subset of females [62]. In addition, it has been reported that both ER-alpha and ER-beta can exert biological activities in the absence of hormones, and in fact, hormones such as estrogen can dampen the ability of ER-alpha and -beta to activate the promoter region of proteinases (i.e., matrix metalloproteinases, MMPs) that can degrade the extracellular matrix (ECM) of connective tissues [68,69,70,71]. ER-beta was found to be more active than ER-alpha on the promoter for some genetic variants of MMP-1 than others [68]. Thus, in the absence of hormones after menopause, there may be a risk for some females to modify the ECM of connective tissues. However, connective tissue throughout the body would be at risk if this mechanism was solely responsible, and that is clearly not the case for the majority of females who do not develop OA. Therefore, there must be additional factors that are also contributing to knee OA risk in susceptible females after menopause.

Such additional factors could relate to maintaining the mechanical integrity of the knee in the subset of at-risk females after menopause. Firstly, the joint could be affected by the development of sarcopenia in muscles affecting knee joint function. In some females, menopause is accompanied by the development of sarcopenia [72,73], and this could also contribute to the loss of integration for the optimal functioning of the knee. Secondly, the “set point” for the knee that evolved during growth and maturation (both pre- and post-puberty) could have been dependent on hormonal factors to maintain knee integrity, and after menopause, such regulation could be altered. As was mentioned earlier, ~20% of young skeletally mature females do not experience changes to joint laxity at different stages of the menstrual cycle [64], so heterogeneity in the regulation of joint biomechanics is established at skeletal maturity. Whether this response/nonresponse phenotype variation is a surrogate biomarker for the development of knee OA later in life is currently unknown.

In spite of the unknowns regarding mechanisms, it is likely that based on the current evidence development of non-PTOA in post-menopausal females should be considered a separate subset of OA patients when considering interventions and treatment options.

### 2.5. OA Risks Associated with Growth and Maturation Variations

For most of evolutionary history, humanoids, including *Homo sapiens,* had an average lifespan of 30–40 years of age. In addition, several of the early variants were shorter than many individuals that exist today. Based on the size of the bunks on the Mayflower, most passengers, even in the 1600s, were shorter than many people today. Male Neanderthals were ~160–175 cm tall, and females 152–156 cm tall, a height that was similar to most Europeans 200 years ago but 12–14 cm shorter than post-WWII Europeans [74]. Thus, a longer lifespan was also accompanied by improved nutrition and altered growth rates during the post-natal period through puberty until skeletal maturity in more recent times. Accelerated growth rates may enhance the risk for compromised integration of the components of the lower extremities, which could pose an increased risk for OA development later in life.

During the early growth phase, the lower extremity motion segments appear to grow in a patterned manner, but considerable individual variation in rate can occur as well as parameters such as varus-valgus features, alignment, and integration of the various components that may grow at different rates. At the onset of puberty, additional growth rate variation can occur as well as the appearance of sex-dependent features. For instance, both males and females land after a jump similarly prior to the onset of puberty but land quite differently post-puberty [75,76,77,78], and this can pose risks for knee injury [79]. At skeletal maturity, the set points for the joints of a lower extremity motion segment are fairly well established. While such set points may provide functioning during adult life, some of the variations may pose a risk during the aging process when the integrative nature becomes less functional, such as accompanying less activity by 60 years of age or the onset of sarcopenia [72,73,80]. Such loss of integration and loss of set point integrity could pose a risk for the development of OA for a subset of individuals of either sex. Some of the variations, such as malalignment [81,82], could exert more potential risk than some other configurations. Thus, gait analysis could also be used to subtype OA patients even further [83].

As the OA-associated with the above-described joint configurations may not be attributed to an event, it could just be described as “idiopathic.” However, OA dependent on such configurations may constitute a separate subtype of OA. To confirm this conclusion will require additional research, perhaps aided by artificial intelligence or machine learning algorithm enhancement of gait patterns at different life stages with a cohort followed longitudinally.

## 3. Implications from Subtyping for Conservative Treatment for Knee OA

### 3.1. Background

**Figure 1:**
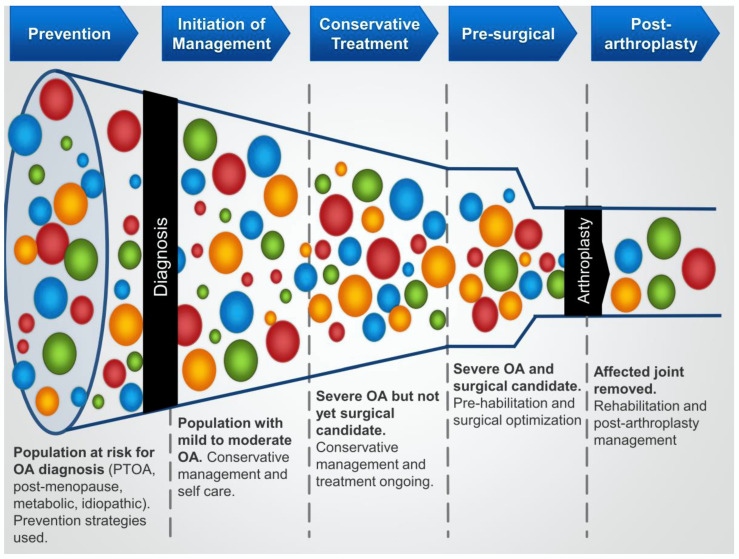
The OA patient “funnel” depicting the ever-increasing bottleneck for joint arthroplasty as the number of patients needing effective conservative care expands. From Hart et al. [11].

Conservative treatment of knee OA, those interventions initiated between the time of diagnosis and perhaps joint replacement surgery, is quite variable and can vary depending on where you live (country, in Canada-the Province, urban vs. rural, etc.), who your main health care provider is (i.e., family physician, physiotherapist, chiropractor, orthopedic surgeon, rheumatologist, etc.), how involved the patient is in their treatment (i.e., shared decision-making), and how informed the patient has become regarding their options (often via the internet). Another variable that can impact patient outcomes is the availability of joint replacement surgery, which, when restricted or in limited supply, can lead to excessive wait times and a prolonged interval of trying to cope with increasing pain and disability (Figure 1) [11]. Thus, there is a real need to develop effective conservative care platforms that consist of integrated and validated interventions to both slow or modify disease progression, improve quality of life via minimizing the impact of the pain and disability, and prepare the patient for optimal outcomes after joint replacement surgery. Unfortunately, in many locations, the availability of some options for conservative treatments are limited; they are not integrated where available, and patients with knee OA are treated as a group without consideration for the subtype phenotype, and individual OA patients may have. Thus, while the phenotyping of OA patients into subgroups depending on the initiating variables or factors that can impact the disease progression is a step forward, it is only the beginning of developing effective conservative care platforms. It is clear that some patients can be considered “responders” to a given treatment while others can be classified as “non-responders” (discussed in [84]). This is likely true for any number of treatment options and can likely be attributed to human heterogeneity [85], as well as disease heterogeneity and structural variation in the disease [86]. Therefore, conservative care should take into account human and disease heterogeneity in an integrated manner and should not remain as a series of “non-effective/effective” interventions that are implemented individually or sequentially until the time for a joint replacement! Unless such a plan is implemented, the backlog of OA patients will continue to overwhelm the health system as the number of patients is expanding (Figure 1).

### 3.2. OA Phenotyping and Conservative Care

The emerging concept that OA, in particular knee OA, is comprised of multiple subsets (Table 1) should not only impact conservative care for knee OA patients with currently available interventions but also spur the development of new subset-specific modalities to treat early OA for patients with those different subtypes of the disease. While addressing subsets of patients is a good step forward, given human heterogeneity, this ultimately could lead to very personalized treatment protocols with precision medicine and perhaps a shift to engaging the patient more effectively in decision-making [87].

The current intervention paradigm for many OA patients after the diagnosis of knee OA is one of increasing “invasiveness” as the condition progresses, following the medical paradigm of “do no harm,” so use the most-mild approaches initially and then increase the invasiveness as the symptoms and degree of disability progresses. As nearly all interventions prior to pre-joint replacement surgical options actually address symptoms and not the associated structural damage to joint components [88], there is a certain fatalism in the treatment paths. Some of this situation is due to a lack of effective clinically approved disease-modifying drugs. However, most of the efforts to develop such candidate drugs often failed to translate from preclinical models or did not consider the variation in potential effectiveness when OA subsets were not taken into account.

Current conservative approaches to assist patients with knee OA include mild analgesics such as oral aspirin and later stronger non-steroidal anti-inflammatories (NSAIDs), bracing (simple sleeves, compartment-lift, mechanical assist), intra-articular injections with glucocorticoids or hyaluronic acid, exercise programs (neuromuscular training programs), and injections with autologous platelet-rich plasma [4,28,84,89]. Some patients also take over-the-counter supplements such as glucosamine and other “joint protective” mixtures [88]. Not all patients respond to the same interventions, with some getting no relief, some short-term relief, and others long-term relief from symptoms [11,84], irrespective of the type of intervention or individual patient characteristics. The basis for such heterogeneity is mainly unknown, but it is known that OA pain relief is unusually susceptible to the placebo effect [90,91], a situation that often makes it difficult to evaluate non-operative treatment [92,93].

For most patients, conservative management is often piecemeal, and various interventions are tried as individual “one-offs” until something is found to offer relief. When such an intervention subsequently fails, the search continues until more invasive options are entertained; such options include pre-joint replacement surgical interventions such as osteotomies [81], drilling/microfracture of the bone [94,95], or debridement alone or in combination with other treatments [96,97,98]. While many of these invasive surgeries offer transient relief for a number of patients, they have not been evaluated for effectiveness regarding knee OA subsets as defined in this review.

In addition to approved interventions, there are also some more “conservative” interventions that remain primarily experimental by regulatory bodies such as the FDA in the USA or Health Canada in Canada. Many of these involve the use of mesenchymal stem or stromal cells derived from a variety of autologous tissues (bone marrow, adipose tissue, synovial tissue, etc.). Such cells may be injected as single-cell preparations or as tissue-engineered constructs (reviewed in [4,28]). Single-cell injections have not proven to be very effective in repairing damaged cartilage, but they have been effective in clinical trials to affect pain and disability [99,100]. However, effectiveness may be negatively influenced by the intra-articular environment, particularly the presence of inflammation [4,101], in spite of their possible immunomodulatory role [102,103]. Some pilot studies with tissue-engineered constructs with autologous synovium-derived mesenchymal stem/stromal cells have indicated the good potential to restore cartilage in cartilage defects prior to the development of overt knee OA [104,105]. Whether such approaches will lead to effective tissue regeneration after the onset of overt knee OA or will require very early intervention before the loss of joint integrity becomes too extreme are currently being considered. However, it may be necessary to factor OA subtypes into the studies as effectiveness likely may require an intra-articular environment that is not too mechanically dysfunctional.

Instead of using stem/stromal cell transfers, it is now emerging that extracellular vesicles (EV) or exosomes released from mesenchymal stem/stromal cells may be as effective in the treatment of conditions such as OA [106,107,108,109]. The EV may stimulate endogenous cells to initiate a reparative process [110]. Thus, if the considerable ongoing research effort shows efficacy in initiating structural repair of tissues damaged by the OA disease process, this may have significant potential in the future. However, if the EV are stimulating endogenous cells to affect repair, then such interventions should be used early, perhaps soon after diagnosis, rather than later in the disease process when many of the potential responder cells have been lost to the disease. Further research on the use of cellular therapies should also consider the intra-articular environment they are being introduced into [4] and when in the disease process they are being applied.

Interestingly, some conservative interventions such as neuromuscular training programs (NMTP) have also been implemented as a precursor or “prehab” in relation to a planned total joint replacement (TJR or TKA-total knee arthroplasty) [11]. The rationale for such a sequence is that improved muscle tone may/should enhance recovery from the surgery and thus facilitate recovery of joint function with rehabilitation post-surgery. Some neuromuscular training programs, such as the GLA:D (Good Living with Arthritis: Denmark), have been well branded and shown to enhance leg function, lower pain management drug use, and decrease disability [111]. Some patients respond so well that they cancel their scheduled TKA surgery. Such success late in the disease process raises the question of why not implement the program at the time of diagnosis. However, not all patients achieve benefits from such exercise programs, mainly resistance exercise protocols, so response patterns are not uniform. Whether responsiveness/non-responsiveness can be impacted by the OA subtype has not been elucidated by clinical trials as yet.

## 4. The Way Forward

Based on the above discussion, there are at least five important factors to consider going forward regarding the conservative treatment of OA, using knee OA as the model system. The first is that a shift from a passive, somewhat fatalistic approach to care be shifted to a more active approach to not only stop progression but initiate repair. This may involve the abandonment of the current medical paradigm and a switch to a paradigm that implements more aggressive non-surgical interventions early in the disease rather than later. Secondly, there should be a focus on OA subtypes regarding existing and future interventions to optimize outcomes (i.e., one size does not fit all). Thirdly, a research effort should be mounted to develop subtype-specific interventions. Fourthly, the conservative interventions most appropriate for specific subtypes should be integrated into an effective platform for individual patients, often using multiple interventions at the same time (i.e., prebiotics and exercise and nutrition for the OA with an obesity phenotype). Lastly, knee OA subsets should be considered when performing genetic analyses of patients with OA. It has been challenging to identify specific genetic contributions through genome-wide scans [112,113,114,115], but perhaps new technologies and assessing subsets of patients (OA subtypes, sex, ethnicity) will yield more precision-based findings.

For instance, it would also be very informative if the pattern of epigenetic changes occurring in tissues and cells from the various subtypes of OA patients were found to be different, thus supporting the subtype designations and potentially providing clues to molecular pathways that contribute to disease. In addition, the use of quantitative mass spectrometry approaches to assess the proteomics of synovial fluid taken early in the disease course could potentially identify subtype-specific biomarkers or identify molecular pathways that could inform intervention development [116,117,118]. While this is an ambiguous plan, failure to implement such a plan will likely fail a large number of existing patients, as well as the expected expansion of the patient population [11].

Of course, the best plan would be to devise programs to minimize the risk of developing knee OA in the first place. Prevention should likely be more effective than treatment. Thus, strategies to prevent OA initiation or minimize risk should be considered a lifespan strategy [119,120]. However, there will likely always be a population that encounter accidents to knee integrity that enhance the risk for OA and others that have genetic and epigenetic risk that may be difficult to prevent. However, having an effective and integrated conservative treatment program that is tailored to the individual patient very early in the disease course could also impact disease progression and disability associated with the condition [121], with particular attention to patients with intersectional disadvantages [122].

## 5. Conclusions

The knee OA patient population is heterogeneous, with different subsets having different characteristics or phenotypes. This heterogeneity can be defined using several different approaches [16,17,18,19], including the categories discussed in this review. The more successful the research community becomes in delineating subtypes of OA, the more understanding will emerge to rationalize the development of effective interventions.

While not as detailed for OA of other joints currently, it is also likely that OA in the hip, shoulder and ankle are also heterogeneous and should be considered such until proven otherwise. Different subsets of OA, based on phenotype, have different initiating features and progression potential and should likely be addressed using more individualized conservative treatment protocols. Such protocols should be integrated throughout the course of the disease.

One common feature of OA of a joint such as the knee is inflammation, but different subsets of patients may vary with regard to inflammation as an initiator of the condition or a consequence of disease progression. However, controlling inflammation should be a critical step in the treatment of knee OA, as the effectiveness of emerging conservative interventions may be compromised if the articular environment is catabolic due to the inflammatory state. Thus, improved approaches to control inflammation and inflammatory processes in joints affected by OA are needed, approaches that avoid some of the side effects of current anti-inflammatory interventions.

Finally, more effort should be expended in developing effective prevention strategies regarding the risk of developing OA across the lifespan. These may be related to education, exercise programs, assessment during growth and maturation, and a more detailed analysis of genetic and epigenetic variables that contribute to risk. As genomic analyses become more sophisticated and identify risk factors for different subtypes of knee OA (or other joints), these contributions can also be incorporated into integrated prevention programs. Until that time, it is also clear that as these tissues of the MSK system subscribe to the “use it or lose it” paradigm, consistent physical activity, good nutrition and avoiding injury risk activities are general principles to follow.

## Figures and Tables

**Table 1:** Subtypes of Knee Osteoarthritis.

Subtype	Initiation	Mechanical Instability ^a^	Inflammation Detected ^b^
PTOA	Joint Injury	YES	YES (due to injury/repair)
Metabolic	Obesity	Not Initially	YES (metabolic syndrome)
Post-Menopausal	Menopause	Not Initially	YES (with tissue degeneration)
Growth/Maturation ^c^	Age Transitions	Not Initially	YES (with tissue degeneration)
(malalignment, muscle)			
Idiopathic ^d^ (cause?)	Aging?/Genetics?	Not Initially	YES (with tissue degeneration)
All likely have some genetic risk associated with subtype and all present with pain and some disability			

^a^ PTOA (Post-Traumatic Osteoarthritis) results from overt damage to joint tissues leading to altered biomechanics and biology. For remaining subtypes, mechanical alterations likely occur as disease progresses. ^b^ For PTOA, inflammation can arise from the initial injury or surgical attempts to repair. For remaining subtypes, the inflammation can be associated with synovitis associated with intra-articular tissue damage. ^c^ Variation in joint tissue integration and/or motion segment (i.e., the leg) characteristics predispose to risk that becomes apparent during aging transitions accompanied by loss of integrated functioning. ^d^ Additional subtypes of OA, not currently identifiable, may exist and are labeled “Idiopathic” or cause unknown.

## Data Availability

Not applicable.

## References

[1] Song Y., Wu Z., Zhao P. (2022). The effects of metformin in the treatment of osteoarthritis: Current perspectives. Front. Pharmacol..

[2] Zhang C., Lin Y., Yan C.H., Zhang W. (2022). Adipokine signaling pathways in osteoarthritis. Front. Bioeng. Biotechnol..

[3] Peck J., Slovek A., Miro P., Vij N., Traube B., Lee C., Berger A.A., Kassem H., Kaye A.D., Sherman W.F. (2021). A comprehensive review of viscosupplementation in osteoarthritis of the knee. Orthop. Rev..

[4] Hart D.A., Nakamura N. (2022). Creating an optimal in vivo environment to enhance outcomes using cell therapy to repair/regenerate injured tissues of the musculoskeletal system. Biomedicines.

[5] Frank C.B., Shrive N.G., Boorman R.S., Lo I.K.Y., Hart D.A. (2004). New perspectives on bioengineering of joint tissues: Joint adaptation creates a moving target for engineering replacement tissues. Ann. Biomed. Eng..

[6] Radin E.L., Burr D.B., Caterson B., Fyhrie D., Brown T., Boyd R.D. (1991). Mechanical determinants of osteoarthrosis. Semin. Arthritis Rheum..

[7] Motta F., Barone E., Sica A., Selmi C. (2022). Inflammaging and osteoarthritis. Clin. Rev. Allergy Immunol..

[8] Heard B.J., Fritzler M.J., Wiley P., McAllister J., Martin L., El-Gabalawy H., Hart D.A., Frank C.B., Krawetz R. (2013). Intraarticular and systemic inflammatory profiles may identify patients with osteoarthritis. J. Rheumatol..

[9] Roskar S., Hafner-Bratkovic I. (2022). The role of inflammasomes in osteoarthritis and secondary joint degeneration diseases. Life.

[10] Gonzalez-Fernandez P., Rodriguez-Nogales C., Jordan O., Allemann E. (2022). Combination of mesenchymal cells and bioactive molecules in hydrogels for osteoarthritis treatment. Eur. J. Pharm. Biopharm..

[11] Hart D.A., Werle J., Robert J., Kania-Richmond A. (2021). Long wait times for knee and hip total joint replacement in Canada: An isolated health problem, or a symptom of a larger problem?. Osteoarthr. Cartil. Open.

[12] Eriksen E.F., Lech O., Nakama G.Y., O’Gorman D.M. (2021). Disease-modifying adjunctive therapy (DMAT) in osteoarthritis-the biological effects of a multi-mineral complex, LithoLexal Joint—A review. Clin. Pract..

[13] Sullivan B., Platt B., Joiner J., Jacobs C., Conley C., Landy D., Stone A.V. (2022). An investigation of Google searches for knee osteoarthritis and stem cell therapy: What are patients searching online. HSS J..

[14] Tong L., Yu H., Huang X., Shen J., Xiao G., Chen L., Wang H., Xing L., Chen D. (2022). Current understanding of osteoarthritis pathogenesis and relevant new approaches. Bone Res..

[15] Allen K.D., Thomas L.M., Golightly Y.M. (2022). Epidemiology of osteoarthritis. Osteoarthr. Cartil..

[16] Yuan C., Pan Z., Zhao K., Li J., Sheng Z., Yao X., Liu H., Zhang X., Yang Y., Yu D. (2020). Classification of four distinct osteoarthritis subtypes with a knee joint tissue transcriptome atlas. Bone Res..

[17] Vongsirinavarat M., Nilmart P., Somprasong S., Apinokul B. (2020). Identification of knee osteoarthritis disability phenotypes regarding activity limitation: A cluster analysis. BMC Musculoskelet. Disord..

[18] Lv Z., Yang Y.X., Li J., Fei Y., Guo H., Sun Z., Lu J., Xu X., Jiang Q., Ikegawa S. (2021). Molecular classification of knee osteoarthritis. Front. Cell Dev. Biol..

[19] Li M., Lan L., Luo J., Peng L., Li X., Zhou X. (2021). Identifying the phenotypic and temporal heterogeneity of knee osteoarthritis: Data from the Ostoearthritis Initiative. Front. Public Health.

[20] Mulugeta A., Eshetie T.C., Kassie G.M., Erku D., Mekonnen A., Lumsden A., Hypponen E. (2022). Association between metabolically different adiposity subtypes and osteoarthritis: A Mendelian randomization study. Arthritis Care Res..

[21] Bay-Jensen A.C., Bihlet A., Byrjalsen J., Andersen J.R., Riis B.J., Christiansen C., Michaelis M., Guehring H., Ladel C., Karsdal M.A. (2021). Serum C-reactive protein metabolite (CRPM) is associated with incidence of contralateral knee osteoarthritis. Sci. Rep..

[22] Wang K., Kim H.A., Felson D.T., Xu L., Kim D.H., Hewitt M.C., Yoshimura N., Kawaquichi H., Lin J., Kan X. (2018). Radiographic knee osteoarthritis and knee pain: Cross-sectional study from five different racial/ethnic populations. Sci. Rep..

[23] Jones G. (2013). Osteoarthritis: Where are we for pain and therapy in 2013?. Aust. Fam. Physician.

[24] Giordano R., Petersen K.K.-S., Arendt-Nielsen L. (2022). The link between epigenetics, pain sensitivity and chronic pain. Scand. J. Pain.

[25] Laigaard J., Karlsen A., Maagaard M., Rosenberg L.K., Creutzburg A., Lunn T.H., Mathiesen O., Overgaard S. (2022). Perioperative prevention of persistent pain after total hip and knee arthroplasty-protocol for two systematic reviews. Acta Anaesthesiol. Scand..

[26] Kluger M.T., Rice D.A., Borotkanics R., Lewis G.N., Somogyi A.A., Barratt D.T., Walker M., McNair P.J. (2022). Factors associated with persistent opioid use 6–12 months after primary total knee arthroplasty. Anaesthesia.

[27] Klasan A., Rice D.A., Kluger M.T., Borotkanics R., McNair P.J., Lewis G.N., Young S.W. (2022). A combination of high preoperative pain and low radiological grade of arthritis is associated with greater intensity of persistent pain 12 months after total knee arthroplasty. Bone Jt. J..

[28] Hart D.A., Nakamura N., Shrive N.G. (2021). Perspective: Challenges presented for regeneration of heterogeneous musculoskeletal tissues that normally develop in unique biomechanical environments. Front. Bioeng. Biotechnol..

[29] Bapat S., Hubbard D., Munjal A., Hunter M., Fulzele S. (2018). Pros and cons of mouse models for studying osteoarthritis. Clin. Transl. Med..

[30] Serra C.I., Soler C. (2019). Animal models of osteoarthritis in small mammals. Vet. Clin. N. Am. Exot. Anim. Pract..

[31] Hart D.A., Martin C.R., Scott M., Shrive N.G. (2021). The instrumented sheep knee to elucidate insights into osteoarthritis development and progression: A sensitive and reproducible platform to integrated research effects. Clin. Biomech..

[32] McCoy A.M. (2015). Animal models of osteoarthritis: Comparisons and key considerations. Vet. Pathol..

[33] Diaz I.R., Viegas C.A., Carvallo P.P. (2018). Large animal models for osteochondral regeneration. Adv. Exp. Med. Biol..

[34] Vakiel P., Shekarforoush M., Dennison C.R., Scott M., Frank C.B., Hart D.A., Shrive N.G. (2020). Stress measurements on the articular cartilage surface using fiber optic technology and in-vivo gait kinematics. Ann. Biomed. Eng..

[35] Vakiel P., Shekarforoush M., Dennison C.R., Scott M., Muench G., Hart D.A., Shrive N.G. (2021). Mapping stresses on the tibial plateau cartilage in an ovine model using in-vivo gait kinematics. Ann. Biomed. Eng..

[36] Shekarforoush M., Vakiel P., Scott M., Muench G., Hart D.A., Shrive N.G. (2020). Relative surface velocity of the tibiofemoral joint and its relation to the development of osteoarthritis after joint injury. Ann. Biomed. Eng..

[37] Richmond S.A., Fukuchi R.K., Ezzat A., Schneider K., Schneider G., Emery C.A. (2013). Are joint injury, sport activity, physical activity, obesity, or occupational activities predictors for osteoarthritis? A systematic review. J. Orthop. Sport. Phys. Ther..

[38] Whittaker J.L., Toomey C.M., Woodhouse L.J., Laremko J.L., Nettel-Aquirre A., Emery C.A. (2018). Association between MRI-defined osteoarthritis, pain, function and strength 3–10 years following knee joint injury in youth sport. Br. J. Sport. Med..

[39] Ezzat A.M., Brussoni M., Masse L.C., Barton C.J., Emery C.A. (2022). New or recurrent knee injury, physical activity, and osteoarthritis beliefs in a cohort of female athletes 2–3 years after ACL reconstruction and matched healthy peers. Sport. Health.

[40] Evers B.J., Van Den Bosch M.H.J., Blom A.B., van den Kraan K.S., Thurlings R.M. (2022). Post-traumatic knee osteoarthritis; the role of inflammation and hemarthrosis on disease progression. Front. Med..

[41] Suarez T., Laudani L., Giombini A., Saraceni V.M., Mariani P.P., Pigozzi F., Macaluso A. (2016). Comparison in joint-position sense and muscle coactivation between anterior cruciate ligament-deficient and healthy individuals. J. Sport Rehabil..

[42] Arhos E.K., Thoma L.M., Grindem H., Logerstedt D., Risberg M.A., Snyder-Mackler L. (2022). Association of quadriceps strength symmetry and surgical status with clinical osteoarthrhritis five years after anterior cruciate ligament rupture. Arthritis Care Res..

[43] Zargi T.G., Drobnic M., Jkoder J., Strazar K., Kacin A. (2016). The effects of preconditioning with ischemic exercise on quadriceps femoris muscle atrophy following anterior cruciate ligament reconstruction: A quasi-randomized controlled trial. Eur. J. Phys. Rehabil. Med..

[44] Tourville T.W., Voight T.B., Choquette R.H., Failla M.J., Endres N.K., Slauterbeck J.R., Beynnon B.D., Toth M.J. (2022). Skeletal muscle cellular contractile dysfunction after anterior cruciate ligament reconstruction contributes to quadriceps weakness at 6-month follow-up. J. Orthop. Res..

[45] Rothrauff B.B., Jorge A., de Sa D., Kay J., Fu F.H., Musahl V. (2020). Anatomic ACL reconstruction reduces risk of post-traumatic osteoarthritis: A systematic review with minimum 10-year follow-up. Knee Surg. Sport. Traumatol. Arthrosc..

[46] Solbak N.M., Heard B.J., Achari Y., Chung M., Shrive N.G., Frank C.B., Hart D.A. (2015). Alterations in Hoffa’s fat pad induced by an inflammatory response following idealized anterior cruciate ligament surgery. Inflamm. Res..

[47] Heard B.J., Barton K.I., Chung M., Achari Y., Shrive N.G., Frank C.B., Hart D.A. (2015). Single intra-articular dexamethasone injection immediately post-surgery in a rabbit model mitigates early inflammatory responses and post-traumatic osteoarthritis-like alterations. J. Orthop. Res..

[48] Heard B.J., Solbak N.M., Chung M., Achari Y., Shrive N.G., Frank C.B., Hart D.A. (2016). The infrapatellar fat pad is affected by injury induced inflammation in the rabbit knee: Use of dexamethasone to mitigate damage. Inflamm. Res..

[49] Heard B.J., Barton K.I., Abubacker S., Chung M., Martin C.R., Schmidt T.A., Shrive N.G., Hart D.A. (2022). Synovial and cartilage responsiveness to peri-operative hyaluronic acid =/_ dexamethasone administration following a limited injury to the rabbit stifle joint. J. Orthop. Res..

[50] Barton K.I., Heard B.J., Sevick J.L., Martin C.R., Shekarforoush S.M.M., Chung M., Achari Y., Frank C.B., Shrive N.G., Hart D.A. (2018). Posttraumatic osteoarthritis development and progression in an ovine model of partial anterior cruciate ligament transection and effect of repeated intra-articular methylprednisolone acetate injections on early disease. Am. J. Sport. Med..

[51] Sieker J.T., Ayturk U.M., Proffen B.L., Weissenberger M.H., Kiapour A.M., Murray M.M. (2016). Immediate administration of intraarticular triamcinolone acetnide after joint injury modulates molecular outcomes associated with early synovitis. Arthritis Rheumatol..

[52] Berenbaum F., Griffin T.M., Liu-Bryan R. (2017). Review: Metabolic regulation of inflammation in osteoarthritis. Arthritis Rheumatol..

[53] Courties A., Sellam J., Berenbaum F. (2017). Metabolic syndrome-associated osteoarthritis. Curr. Opin. Rheumatol..

[54] Zapata-Linares N., Eymard F., Berenbaum F., Houard X. (2021). Role of adipose tissues in osteoarthritis. Curr. Opin. Rheumatol..

[55] Batushansky A., Zhu S., Komaravolu R.K., South S., Mehta-D’souza P., Griffin T.M. (2022). Fundamentals of OA: An initiative of osteoarthritis and cartilage. Obesity and metabolic factors in OA. Osteoarthr. Cartil..

[56] Nemet M., Blazin T., Milutinovic S., Cebovic T., Stanojevic D., Svorcan J.Z. (2022). Association between metabolic syndrome, its components, and knee osteoarthritis in premenopausal and menopausal women: A pilot study. Cureus.

[57] Collins K.H., Herzog W., MacDonald G.Z., Reimer R.A., Rios J.L., Smith I.C., Zernicke R.F., Hart D.A. (2018). Obesity, metabolic syndrome, and musculoskeletal disease: Common inflammatory pathways suggest a central role for loss of muscle integrity. Front. Physiol..

[58] Collins K.H., Hart D.A., Seerattan R.A., Reimer R.A., Herzog W. (2018). High-fat/high-sucrose diet-induced obesity results in joint-specific development of osteoarthritis-like degeneration in a rat model. Bone Jt. Res..

[59] Rios J.L., Bomhof M.R., Reimer R.A., Hart D.A., Collins K.H., Herzog W. (2019). Protective effect of prebiotic and exercise intervention on knee health in a rat model of diet-induced obesity. Sci. Rep..

[60] Fortuna R., Hart D.A., Sharkey K.A., Schachar R.A., Johnston K., Reimer R.A. (2021). Effect of a prebiotic supplement on knee joint function, gut microbiota, and inflammation in adults with co-morbid obesity and knee osteoarthritis: Study protocol for a randomized controlled trial. Trials.

[61] Berenbaum F., Walker C. (2020). Osteoarthritis and inflammation: A serious disease with overlapping phenotypic patterns. Postgrad. Med..

[62] Hart D.A. (2022). Sex differences in biological systems and the conundrum of menopause: Potential commonalities in post-menopausal disease mechanisms. Int. J. Mol. Sci..

[63] Boyan B.D., Hart D.A., Enoka R.M., Nicolella D.P., Resnick E., Berkley K.L., Sluka K.A., Kwoh C.K., Tosi L.L., O’Conner M.I. (2013). Hormonal modulation of connective tissue homeostasis and sex differences in risk for osteoarthritis of the knee. Biol. Sex Diff..

[64] Park S.K., Stefanyshyn D.J., Loitz-Ramage B., Hart D.A., Ronsky J.L. (2009). Changing hormone levels during the menstrual cycle affect knee laxity and stiffness in healthy female subjects. Am. J. Sport. Med..

[65] Chu S.R., Boyer E.H., Beynnon B., Segal N.A. (2019). Pregnancy results in lasting changes in knee joint laxity. PM&R.

[66] Sciore P., Frank C.B., Hart D.A. (1998). Identification of sex hormone receptors in human and rabbit ligament of the knee by reverse transcription-polymerase chain reaction: Evidence that receptors are present in tissue from both male and female subjects. J. Orthop. Res..

[67] Liu S.H., al-Shaikh R., Ponossian V., Yang R.S., Nelson S.D., Soleiman N., Fineman G.A., Lane J.M. (1996). Primary immunolocalization of estrogen and progesterone target cells in the human anterior cruciate ligament. J. Orthop. Res..

[68] Achari Y., Lu T., Hart D.A. (2008). Polymorphisms in the promoter regions for human MMP-1 and MMP-13 lead to differential responses to the alpha and beta isoforms of estrogen receptor and their ligand in vitro. Biochim. Biophys. Acta.

[69] Achari Y., Lu T., Katzenellenbogen B.S., Hart D.A. (2009). Distinct roles for AF-1 and -2 of ER-alpha in regulation of MMP-13 promoter activity. Biochim. Biophys. Acta.

[70] Lu T., Achari Y., Sciore P., Hart D.A. (2006). Estrogen receptor alpha regulates metalloproteinase-13 promoter activity primarily through the AP-1 transcriptional regulatory site. Biochim. Biophys. Acta.

[71] Lu T., Achari Y., Rattner J.B., Hart D.A. (2007). Evidence that estrogen receptor beta enhances MMP-13 promoter activity in HIG-cells and that this enhancement can be influenced by ligands and involves specific promoter sites. Biochem. Cell Biol..

[72] Kaji H. (2013). Linkage between muscle and bone: Common catabolic signals resulting in osteoporosis and sarcopenia. Curr. Opin. Clin. Nutr. Metab. Care.

[73] Agostini D., Donati S.Z., Lucertini F., Annibalini G., Gervasi M., Marini C.F., Piccoli G., Stocchi V., Barbieri E., Sestili P. (2018). Muscle and bone health in postmenopausal women: Role of protein and vitamin D supplementation combined with exercise training. Nutrients.

[74] Helmuth H. (1998). Body height, body mass and surface area of the Neanderthals. Z. Für Morphol. Anthropol..

[75] Quatman C.E., Ford K.R., Myer G.D., Hewett T.E. (2006). Maturation leads to gender differences in landing force and vertical jump performance: A longitudinal study. Am. J. Sport. Med..

[76] Ford K.R., Shapiro R., Myere G.D., Van Den Bogert A.J., Hewett T.E. (2010). Longitudinal sex differences during landing in knee abduction in young athletes. Med. Sci. Sport. Exerc..

[77] Carson D.W., Ford K.R. (2011). Sex differences in knee abduction during landing: A systematic review. Sport. Health.

[78] Hewett T.E. (2015). Longitudinal increases in knee abduction moments in females during adolescent growth. Med. Sci. Sport. Exerc..

[79] Hewett T.E., Roewer B., Ford K., Myer G. (2015). Multicenter trial of motion analysis for injury risk prediction: Lessons learned from prospective longitudinal large cohort combined biomechanical -epidemiological studies. Braz. J. Phys. Ther..

[80] De Souza L.F., Danielewicz A.L., Rech C.R., d’Orsi E., Mendonca V.A., Lacerda A.C.R., de Avelar N.C.P. (2022). How much time in sedentary behavior is associated with probable sarcopenia in older adults?. Geriatr. Nurs..

[81] Ollivier B., Berger P., Depuydt C., Vandenneucjer H. (2021). Good long-term survival and patient-reported outcomes after high tibial osteotomy for medial compartment osteoarthritis. Knee Surg. Sport. Traumatol. Arthrosc..

[82] Diaz C.C., Lavoie-Gagne O.Z., Knapik D.M., Korrapati A., Chala J., Forsythe B. (2022). Outcomes of distal femoral osteotomy for valgus malalignment: A systematic review and meta-analysis of closing wedge versus opening wedge techniques. Am. J. Sport. Med..

[83] Robbins S.M., Pelletier J.-P., Abram F., Boily M., Antoniou J., Martineau P.A., Morelli M., Martel-Pelletier J. (2021). Gait risk factors for disease progression differ between non-traumatic and post-traumatic knee osteoarthritis. Osteoarthr. Cartil..

[84] Kydd A.S.R., Hart D.A. (2020). Efficacy and safety of platelet-rich plasma injections for osteoarthritis. Curr. Treat. Options Rheumatol..

[85] Vina E.R., Tsoukas P.H., Abdollahi S., Mody N., Roth S.C., Redford A.H., Kwoh C.K. (2022). Racial and ethnic differences in the pharmacologic management of osteoarthritis: Rapid systematic review. Ther. Adv. Musculoskelet. Dis..

[86] Roemer F.W., Jarraya M., Collins J.E., Kwoh C.K., Hayashi D., Hunter D.J., Guermazi A. (2022). Structural phenotypes of knee osteoarthritis: Potential clinical and research relevance. Skeletal. Radiol..

[87] Caneiro J.P., O’Sullivan P.B., Roos E.M., Smith A.J., Choong P., Dowsey M., Hunter D.J., Kemp J., Rodriguez J., Lohmander S. (2020). Three steps to changing the narrative about knee osteoarthritis care: A call to action. Br. J. Sport. Med..

[88] Overton C., Nelson A.E., Neogi T. (2022). Osteoarthritis treatment guidelines from six professional societies: Similarities and differences. Rheum. Dis. Clin. N. Am..

[89] Lana J.F., Macedo A., Ingrao I.L.G., Huber S.C., Santos G.S., Santana M.H.A. (2019). Leukocyte-rich PRP for knee osteoarthritis: Current concepts. J. Clin. Orthop. Trauma.

[90] Zhang W. (2019). The powerful placebo effect in osteoarthritis. Clin. Exp. Rheumatol..

[91] Fazeli M.S., McIntyre L., Huang Y., Chevalier X. (2022). Intra-articular placebo effect in the treatment of knee osteoarthritis: A survey of the current clinical evidence. Ther. Adv. Musculoskelet. Dis..

[92] Vannabouathong C., Bhandari M., Bedi A., Khanna V., Yung P., Shetty V., Khan M. (2018). Nonoperative treatments of knee osteoarthritis: An evaluation of treatment characteristics and the intra-articular placebo effect: A systematic review. JBJS Rev..

[93] Thorlund J.B., Simic M., Pihl K., Berthelsen D.B., Day R., Koes B., Juhl C.B. (2022). Similar effects of exercise therapy, nonsteroidal anti-inflammatory drugs, and opioids for knee osteoarthritis pain: A systematic review with network meta-analysis. J. Orthop. Sport. Phys. Ther..

[94] Brumat P., Kunsic O., Novak S., Slokar U., Psenica J., Topolovec M., Mihalic R., Trebse R. (2022). The surgical treatment of osteoarthritis. Life.

[95] Hoburg A., Niemyer P., Laute V., Zinser W., Becher C., Kolombe T., Fay J., Pietsch S., Kuzma T., Widuchowski W. Sustained superiority in KOOS subscores after matrix-associated chondrocyte implantation using spheroids compared to microfracture. Knee Surg. Sport. Traumatol. Arthrosc..

[96] Tirtosuharto H., Wiratnaya G.E., Astawa P. (2022). Adjunctive platelet-rich plasma and hyaluronic acid injection after arthroscopic debridement in Kellgren-Lawrence grade 3 and 4 knee osteoarthritis. World J. Orthop..

[97] Lu M., Jin Y. (2022). Efficacy evaluation of the combined platelet-rich plasma and hyaluronic acid after arthroscopic joint debridement in treating knee osteoarthritis. Scanning.

[98] Lin T., Liu Z., Ji W., Zhang P. (2022). Effects of knee debridement with flurbiprofen on knee function, inflammatory levels, and bone metabolism activity in patients with knee osteoarthritis. Comput. Math. Methods Med..

[99] Yokota N., Lyman S., Hanai H., Shimomura K., Ando W., Nakamura N. (2022). Clinical safety and effectiveness of adipose-derived stromal cell vs stromal vascular fraction injection for treatment of knee osteoarthritis: 2-year results of parallel single-arm trials. Am. J. Sport. Med..

[100] Kim K., Lee W.S., Kim J.H., Bae J.K., Jin W. (2022). Safety and efficacy of the intra-articular injection of mesenchymal stem cells for the treatment of osteoarthritic knee: A 5-year follow-up study. Stem Cells Transl. Med..

[101] Ferracini R., Alessio-Mazzola M., Sonzogni B., Stambazzi C., Ursino C., Roato I., Mussano F., Bistolfi A., Furlan A., Godio L. (2022). Age and synovitis affect the results of the treatment of knee osteoarthritis with microfragmented autologous fat tissue. Knee Surg. Sport. Traumatol. Arthrosc..

[102] Kwon D.G., Kim M.K., Jeon Y.S., Nam Y.C., Park J.S., Ryu D.J. (2022). State of the art: The immunomodulatory role of MSCs for osteoarthritis. Int. J. Mol. Sci..

[103] Wang S., Lei B., Zhang E., Gong P., Gu J., He L., Han L., Yuan Z. (2022). Targeted therapy for inflammatory diseases with mesenchymal stem cells and their derived oxosome: From basics to clinics. Int. J. Nanomed..

[104] Shimomura K., Yasui Y., Koizumi K., Chijimatsu R., Hart D.A., Yonetani Y., Ando W., Nishii T., Kanomoto T., Horibe S. (2018). First-in-human pilot study of implantation of a scaffold-free tissue-engineered construct generated from autologous synovial mesenchymal stem cells for repair of knee chondral lesions. Am. J. Sport. Med..

[105] Shimomura K., Hamada H., Hart D.A., Ando W., Nishii T., Tratting S., Neher S., Nakamura N. (2021). Histological analysis of cartilage defects repaired with an autologous human stem cell construct 48 weeks postimplantation reveals structural details not detected by T2-mapping MRI. Cartilage.

[106] D’Arrigo D., Roffi A., Cucchiarini M., Moretti M., Candrian C., Filardo G. (2019). Secretome and extracellular vesicles as new biological therapies for knee osteoarthritis: A systematic review. J. Clin. Med..

[107] Tang S., Chen P., Zhang H., Weng H., Fang Z., Chen C., Peng G., Gao H., Hu K., Chen J. (2021). Comparison of curative effect of human umbilical cord-derived mesenchymal stem cells and their small extracellular vesicles in treatment osteoarthritis. Int. J. Nanomed..

[108] Jeyaraman M., Muthu S., Shehabaz S., Jeyaraman N., Rajendran R.L., Hong C.M., Nallakumarasamy A., Packkyarathinam R.P., Sharma S., Ranjan R. (2022). Current understanding of MSC-derived exosomes in the management of knee osteoarthritis. Exp. Cell Res..

[109] Pandey V., Madi S., Gupta P. (2022). The promising role of autologous and allogenic mesenchymal stromal cells in managing knee osteoarthritis. What is beyond mesenchymal stromal cells?. J. Clin. Orthop. Trauma.

[110] Phelps J., Leonanrd C., Shah S., Krawetz R., Hart D.A., Duncan N.A., Sen A. (2022). Production of mesenchymal progenitor cell-derived extracellular vesicles in suspension bioreactors for use in articular cartilage repair. Stem Cells Trans. Med..

[111] Baumbach L., Gronne D.T., Moller N.C., Skou S.T., Roos E.M. (2022). Changes in physical activity and the association between pain and physical activity-a longitudinal analysis of 17,454 patients with knee or hip osteoarthritis from the GLA:D registry. Osteoarthr. Cartil..

[112] Panoutsopoulou K., Zeggini E. (2013). Advances in osteoarthritis genetics. J. Med. Genet..

[113] Zengini E., Finan C., Wilkinson J.M. (2016). The genetic epidemiological landscape of hip and knee osteoarthritis: Where are we now and where are we going?. J. Rheumatol..

[114] Cai Z., Long T., Zhao Y., Lin R., Wang Y. (2022). Epigenetic regulation in knee osteoarthritis. Front. Genet..

[115] Wang T., Liang Y., Li H., Li H., He Q., Xue Y., Shen C., Zhang C., Xiang J., Ding J. (2016). Single nucleotide polymorphisms and osteoarthritis: An overview and a meta-analysis. Medicine.

[116] Lee Y.R., Briggs M.T., Condina M.R., Puddy H., Anderson P.H., Hoffmann P., Kuliwaba J.S. (2020). Mass spectrometry imaging as a potential tool to investigate human osteoarthritis at the tissue level. Int. J. Mol. Med..

[117] Timur U.T., Jahr H., Anderson J., Green D.C., Means P.J., Smagul A., van Rhijn L.W., Peffers M.J., Welting T.J.M. (2021). Identification of tissue-dependent proteins in knee OA synovial fluid. Osteoarthr. Cartil..

[118] Brophy R.H., Cai L., Zhang Q., Townsend R.T., Rai M.F. (2022). Proteomic profile of synovial fluid in patients with anterior cruciate ligament tears. Am. J. Sport. Med..

[119] Whittaker J.L., Runhaar J., Bierma-Zeinstra S., Roos E.M. (2021). A lifespan approach to osteoarthritis prevention. Osteoarthr. Cartil..

[120] Roos E.M., Arden N.K. (2016). Strategies for the prevention of knee osteoarthritis. Nat. Rev. Rheumatol..

[121] Whittaker J.L., Roos E.M. (2019). A pragmatic approach to prevent post-traumatic osteoarthritis after sport or exercise-related joint injury. Best Pract. Res. Clin. Rheumatol..

[122] Peat G., Yu D., Gronne D.T., Marshall M., Skou S.T., Roos E.M. (2022). Do patients with intersectional disadvantage have poorer outcomes form osteoarthritis management programs? A tapered balancing study of patient outcomes from the good life with osteoarthritis in Denmark program. Arthritis Care Res..

